# Mobility analysis of a posterior sacrospinous fixation using a finite element model of the pelvic system

**DOI:** 10.1371/journal.pone.0299012

**Published:** 2024-03-21

**Authors:** Marine Lallemant, Andres Arteaga Shimojyo, Olivier Mayeur, Rajeev Ramanah, Chrystèle Rubod, Yohan Kerbage, Michel Cosson

**Affiliations:** 1 Service de Gynécologie Obstétrique, Pôle Mère-Femme, Centre Hospitalier Universitaire Jean Minjoz, Besançon, France; 2 Université Lille, CNRS, Centrale Lille, UMR 9013—LaMcube—Laboratoire de Mécanique, Multiphysique, Multiéchelle, F-59000, Lille, France; 3 Laboratoire de Nanomédecine, Imagerie et Thérapeutiques, INSERM E4 4662, Université de Franche-Comté, Besançon, France; 4 CHU Lille, Service de Chirurgie Gynécologique, F-59000, Lille, France; 5 Faculté de médecine, Université Lille Nord de France, F-59000, Lille, France; University of Zaragoza, SPAIN

## Abstract

**Introduction and hypothesis:**

In order to improve the knowledge POP physiopathology and POP repair, a generic biomechanical model of the female pelvic system has been developed. In the literature, no study has currently evaluated apical prolapse repair by posterior sacrospinous ligament fixation using a generic model nor a patient-specific model that personalize the management of POP and predict surgical outcomes based on the patient’s pre-operative Magnetic Resonance Imaging. The aim of our study was to analyze the influence of a right and/or left sacrospinous ligament fixation and the distance between the anchorage area and the ischial spine on the pelvic organ mobility using a generic and a patient-specific Finite Element model (FEM) of the female pelvic system during posterior sacrospinous ligament fixation (SSF).

**Methods:**

Firstly, we used a generic 3D FEM of the female pelvic system previously made by our team that allowed us to simulate the mobility of the pelvic system. To create a patient-specific 3D FEM of the female pelvic system, we used a preoperative dynamic pelvic MRI of a 68 years old woman with a symptomatic stage III apical prolapse and cystocele. With these 2 models, a SSF was simulated. A right and/or left SSF and different distances between the anchorage area and the ischial spine (1 cm, 2 cm and 3 cm.) were compared. Outcomes measures were the pelvic organ displacement using the pubococcygeal line during maximal strain: Ba point for the most posterior and inferior aspect of the bladder base, C point the cervix’s or the vaginal apex and Bp point for the anterior aspect of the anorectal junction.

**Results:**

Overall, pelvic organ mobility decreased regardless of surgical technique and model. According to the generic model, C point was displaced by 14.1 mm and 11.5 mm, Ba point by 12.7 mm, and 12 mm and Bp point by 10.6 mm and 9.9 mm after left and bilateral posterior SSF, respectively. C point was displaced by 15.4 mm and 11.6 mm and Ba point by 12.5 mm and 13.1mm when the suture on the sacrospinous ligament was performed at 1 cm and 3 cm from the ischial spine respectively (bilateral posterior SSF configuration). According to the patient-specific model, the displacement of Ba point could not be analyzed because of a significative and asymmetric organ displacement of the bladder. C point was displaced by 4.74 mm and 2.12 mm, and Bp point by 5.30 mm and 3.24 mm after left and bilateral posterior SSF respectively. C point was displaced by 4.80 mm and 4.85 mm and Bp point by 5.35 mm and 5.38 mm when the suture on the left sacrospinous ligament was performed at 1 cm and 3 cm from the ischial spine, respectively.

**Conclusion:**

According to the generic model from our study, the apex appeared to be less mobile in bilateral SSF. The anchorage area on the sacrospinous ligament seems to have little effect on the pelvic organ mobilities.

**Trial registration:**

ClinicalTrials.gov Identifier: NCT04551859.

## Introduction

A pelvic organ prolapse is the consequence of an excessive mobility of one or more pelvic organs due to an alteration of their suspension and support systems. Pelvic organ prolapse (POP) is a common condition in women. Although rarely responsible for significant morbidity and mortality, it can severely affect quality of life. The probability of undergoing surgery for prolapse at the age of 80 is 11.1% [1]. Posterior sacrospinous ligament fixation (SSF) according to Richter is an autologous surgery that can be proposed in apical prolapse repair [2]. This surgery restores support to the uterus or the vaginal vault using stitches placed on the sacrospinous ligament. The recurrence rate after SSF ranges from 11% to 35% [3–5].

Classically, this surgery is performed unilaterally [6]. But, unilateral or bilateral fixation remains controversial. Some authors have shown that the bilateral approach could not provide satisfactory midline support [7]. The vaginal apex could not be supported and would therefore be more vulnerable to intrapelvic pressure. Other authors state that bilateral vaginal fixation ensures uniform distribution of traction forces on the vagina and that its horizontal anatomical axis could be conserved. Thus, bilateral fixation could reduce postoperative dyspareunia, postoperative recurrences and cause to fewer intestinal symptoms [8–10]. Biomechanical study of bilateral sacrospinous ligament fixation has never been carried out in the literature.

Moreover, according to several studies, the anchorage location on the sacrospinous ligament for SSF differs from 1.5 cm to 3 cm medial to the ischial spine [9,11–13]. Giraudet et al. demonstrated that the medial part of the sacrospinous ligament (between 16 and 32 mm from the ischial spine) was the most appropriate for the placement of the suture during SSF procedures, in order to avoid nerve and vessel injuries [14]. No study compared the different anchorage points from a clinical or biomechanical point of view.

In this context, we hypothesize that numerical simulation using the Finite Element (FE) method could help reduce recurrences by analyzing the mobility of the pelvic system following different kinds of SSF and understanding the phenomena involved in this complex equilibrium [15–18].

In order to improve the knowledge of this complex system, a biomechanical model of the female pelvic system allowing to simulate and study the mobilities of the pelvic organs from pelvic MRI (Magnetic Resonance Imaging) has been developed [16,17]. The results obtained on a healthy patient come from the team’s previous studies, which linked the patient’s actual mobility [19,20] to the digital twin [21,22]. These studies validated the model by comparing displacement fields analyzed in dynamic MRI and finite element simulations. This generic model was initially used to understand pelvic organ hypermobility in POP [18,23]. Few studies have focused on surgical repair of POP [24]. But, no study has currently evaluated the mobility and stresses of the pelvic organs before and after apical prolapse repair by posterior sacrospinous ligament fixation. Moreover, these studies were not patient-specific [25,26]. The originality of this work comes also from the fact that we customized the model to become patient-specific. The purpose of this patient-specific model would be to personalize management of POP and predict surgical outcomes based on the patient’s pre-operative MRI.

The aim of our study was to analyze the influence of a right and/or left sacrospinous ligament fixation and the distance between the anchorage zone and the ischial spine on the pelvic organ mobility using a generic and a patient-specific Finite Element model of the female pelvic system during posterior sacrospinous ligament fixation.

## Materials and methods

### Generic model

**Fig 1:**
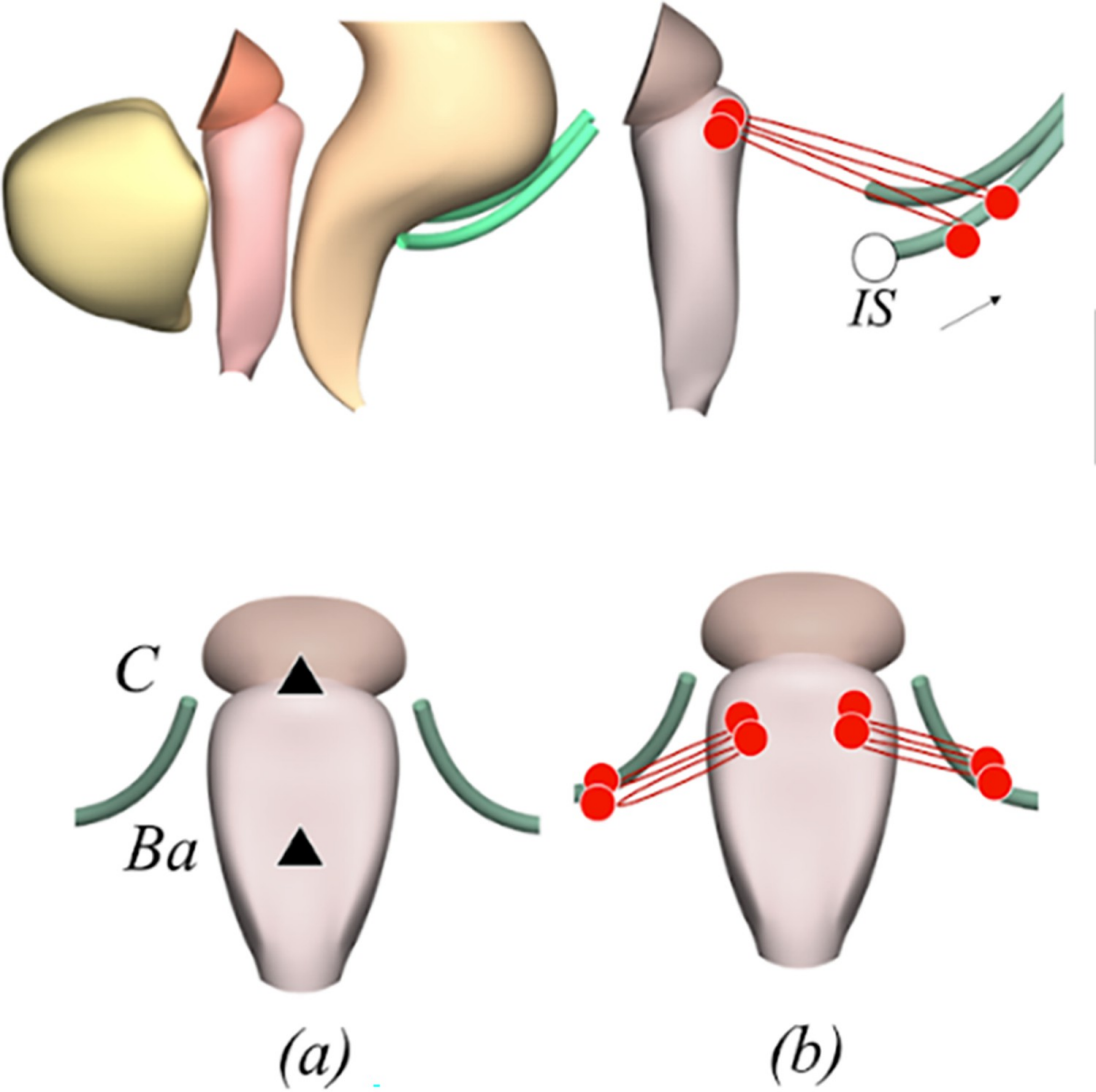
Simulation of posterior sacrospinous ligament fixation with the generic model. (a) Generic model of the pelvic organs (front and sagittal views), (b) Surgical modeling of right and left posterior sacrospinous ligament fixation (front and sagittal views).

For the creation of the generic model, we used a 3D Finite element model of the female pelvic system previously made by our team [18,25–27]. First, we created a geometric model based on an MRI of a woman aged 30 years without prolapse or other pelvic pathology on clinical examination. Pelvic MRI was performed supine, with high-resolution T1- and T2-weighted coronal, axial and sagittal sequences (slice thickness, 3 mm; interval, 3 mm, voxel size, 0.48–0.72 mm; resolution, 512 9 512). Only T2 static sequences were used to construct the FE model. The different anatomical structures selected were: uterus, vagina, bowel, pubocervical fascia, endopelvic fascia, ATFP, ATLA, uterosacral ligament, cardinal ligament and levator ani muscle. The segmentation of the anatomical structures on the MRI was carried out using AVIZO software. The obtained models were then smoothed and corrected using CATIA software to obtain smooth surfaces and the most realistic shapes. Finally, the model represented the structure of woman’s with normal support (without any pathological condition). Since prolapse results from a defect in the anatomical suspension and support system [28,29], our aim was to analyze organ mobility during surgical procedures. The anatomical support system dysfunction was generated by disabling the broad and the round ligaments. The interfaces between organs such as between the rectum and the vagina or between the bladder and the vagina were conserved. The generic model was strictly symmetrical. Then, while using the CAD software CATIA V5 (Dassault Systems), a posterior sacrospinous ligament fixation was manually modeled (Fig 1). An implant was attached from the vaginal apex to the sacrospinous ligament. The constitutive material of sutures was made of isotropic elastic material while the fixation of sacrospinous ligament was implemented with two sutures between the vaginal apex and the sacrospinous ligament. In the absence of precision, the suture was placed 2 cm away from de ischial spine. A right and/or left posterior SSF and different distances between the anchorage zone and the ischial spine (1 cm, 2 cm and 3 cm in a bilateral posterior SSF configuration) were also simulated (Fig 1).

### Patient-specific model

We conducted a prospective preliminary study in the gynecologic surgery departments of Besançon and Lille (France) called BiomecaRichter since October 2021. The protocol was approved by IDF VII Ethics Committee (reference number 2020-A01439-30) and was registered as a clinical trial (NCT04551859) before the initiation of the study recruitment. Inclusion criteria were women ≥ 18 years old who underwent a posterior sacrospinous ligament fixation (SSF) for at least a stage II apical prolapse according to the POP-Q classification. Exclusion criteria were a history of abdominal, pelvic, vaginal or vulvar surgery except hysterectomy, a concomitant urinary incontinence surgery, MRI contraindication, legal incapacity or limited legal capacity. In this work, we studied the case of the first included woman.

According to the study protocol, after accepting the surgeon’s proposal to perform a SSF, participation in this study was proposed to the patient. Consent was informed and a written consent was obtained. The management or the course of the surgery was not changed.

The first included patient was a 68 years old woman without comorbidities. She presented a symptomatic stage III apical prolapse and a stage III cystocele according to the POP-Q classification. Before surgery, a dynamic pelvic MRI was performed. Pelvic MRI was performed supine, with high-resolution T1- and T2-weighted coronal, axial and sagittal sequences. In the following two months, the women underwent a left posterior SSF. The technique of posterior sacrospinous fixation according to Richter began with a vaginal hysterectomy [13]. Then, the posterior vaginal wall was exposed and infiltrated (mix of 30 mL Xylocaine 1% and 30 mL of isotonic saline). A posterior colpotomy was performed. The left pararectal fossa was dissected. The vaginal apex was attached to the left sacrospinous ligament at 2 cm from the ischia spine [11,14]. Then, the vagina was partially closed. The sacrospinous ligament fixation was tightened, and the vagina was completely closed. Post-operative follow-up appointments were scheduled as usual. The patient went back home on the second postoperative day.

Regarding the patient specific model for our study, we used a 3D Finite Element model generation procedure of the female pelvic system, already developed by our team [25]. The following steps were necessary to generate this model (Table 1).

**Table 1:** Steps to obtain a patient-specific finite element model of pelvic organ prolapse using the patient’s preoperative’ MRI.

Steps	How?	With?
Step 1: Creation of a geometrical model	Segmentation of anatomical structures on the MRI	3D Slicer software
Step 2: Smoothing surfaces	Smoothing tool	3D Slicer software
Step 3: Geometric CAD model	• Import of the geometrical model into a geometric CAD model • Manual reconstruction of a mathematical model defined by control points, 3D splines and generation of surfaces and volumes to represent the entire system	CATIA V5-6R2013 software
Step 4: Surgery modulization for simulation	• Creation of the parametric surgical technique • Connection between prosthetic element and patient	CATIA V5-6R2013 software
Step 5: Finite Element Model	• Import of the Patient specific geometric CAD model in the Finite Elements (FE) computing software • Organ modeling using Finite Element (appendix1) • Implementation of Mechanical properties of each organ and biological tissues (appendix1) • Definition of contact interaction between parts	Abaqus/ CAE v 6.14–2 (Dassault Systèmes Simulia) softwares
Step 6: Mobility measurements	Simulation of the patient specific organ displacement seen on the MRI analysis and estimate the force exerted by the abdominal pressure on the pelvic organs	Finite Element Analysis

CAD: Computer-aided design; MRI: Magnetic resonance imaging.

**Fig 2:**
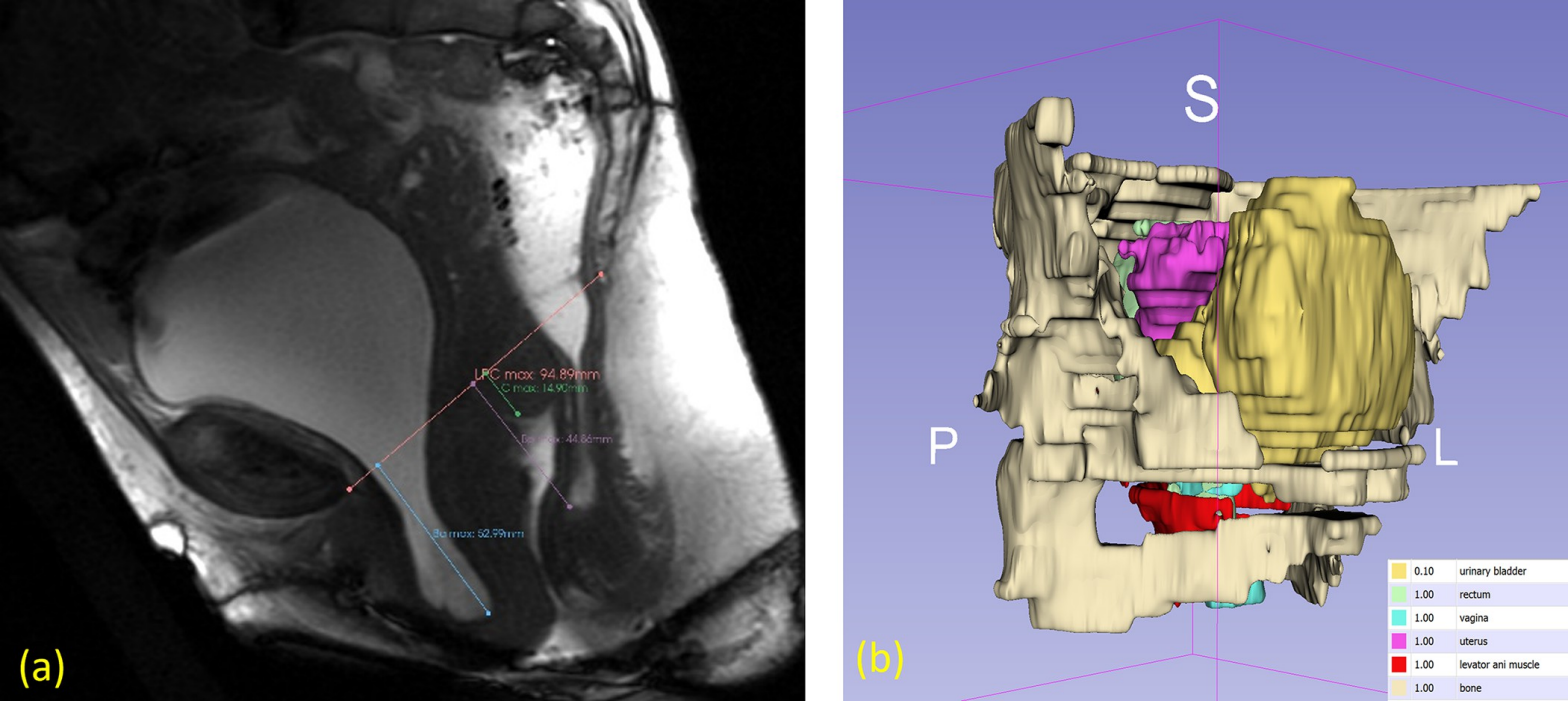
Construction of a geometric model based on the preoperative patient-specific Magnetic Resonance Imaging (MRI). (a) Pelvic organ displacement measurements (mm) on the sagittal section of the dynamic MRI under maximum strain using the pubococcygeal line (PCL) method [30]: Pubococcygeal line (pink), anterior compartment (Ba max point), cervix (C max point), posterior compartment (Bp max point). (b) 3D reconstruction of the pelvic system from the patient-specific preoperative MRI.

First, we created a geometrical model based on the preoperative MRI (Magnetic Resonance Imaging) of the patient to later specify the position of the different anatomical structures. Anatomical structures on the MRI were segmented using 3D Slicer software at this turn. The highlighted and segmented organs were the pelvic bone, the vagina, the uterus, the bladder, the rectum and the levator ani muscle (Fig 2). The segmentations obtained were smoothened with the smoothing tool from 3D Slicer software to obtain smoother surfaces and more realistic forms. All the anatomical structures mentioned were exported as STL files as a single geometric model to keep the same reference system and patient specific organ placement.

**Fig 3:**
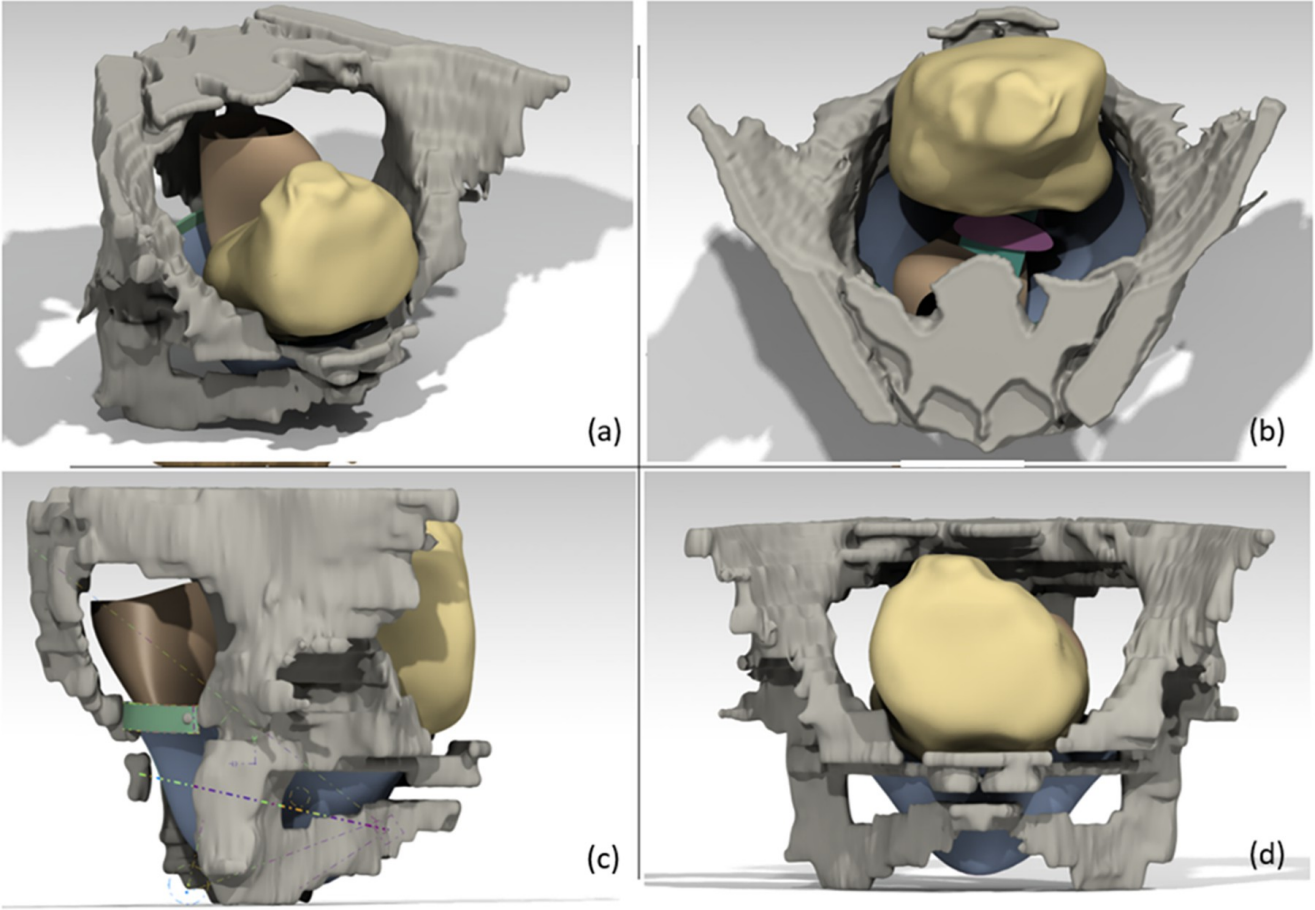
Surface reconstruction of the patient-specific model: Top view (a), posterior view (b), sagittal view (c) and front view (d).

This geometric model was then imported into the geometric CAD model with software CATIA V5-6R2013, compatible with the numerical simulation software Abaqus CAE 2022 [25,26]]. Then, we modeled a total hysterectomy and recreated the pubocervical fascia, endopelvic fascia and ATFP by using the CAD software CATIA V5-6R2013. The geometric model corresponds to an initial state and represents the anatomical shape in a resting position. The CAD process therefore relies on the manual reconstruction of a mathematical model defined by control points, 3D splines and generation of surfaces and volumes to represent the entire system (Fig 3). This step enables controlled geometry to be imported into numerical simulation software and guarantees the viability of the Finite Element mesh and number of elements for each structure. The pelvic floor was modeled through a representative surface sustaining organs and equivalent to the pelvic muscles. Particular attention was also taken to create the parametric model of the surgical technique on this pre-operative geometry. In this way, we were able to model the support elements used to simulate the surgery. This meant that the prosthetic elements were linked to the vagina and sacrospinous ligaments at the insertion nodes. The position of the filament on the vagina was not studied. On the other side, the position on sacrospinous ligament was considered as an input parameter to study the influence of the attachment point (position from the ischial spine in cm). To ensure the perfect connection between anatomical structures and implant fixation, suture points were implemented as a tie contact interaction to constrain the sacrospinous ligament fixation on the apical point of the vagina and the sacrospinous ligament. The ligament fixation filaments were created as constant fil with uniform section. Thanks to this protocol, a right and/or left posterior SSF and different distances between the anchorage zone and the ischial spine (1 cm, 2 cm and 3 cm in a bilateral posterior SSF configuration) could be simulated.

This patient specific geometric CAD model was then imported in the Finite Elements (FE) computing software Abaqus/ CAE v 6.14–2 (Dassault Systèmes Simulia) based on a standard protocol described in the literature [25]. Organs were modeled using Finite Element, such as shell elements with constant thickness and tetrahedral or quad elements when required. Mechanical properties of each organ and biological tissue were implemented at this step [21]. As patient-specific material properties are unknown, we have taken the average mechanical properties of the system already published by our team in the literature. This study focused on organ-specific patient geometry. However, in modeling mechanical behavior, we considered hyperelasticity to investigate large transformations and displacements for the vagina, the bladder and the rectum. Their corresponding hyperelastic properties and C0 Yeoh coefficients were set as 0.111 MPa, 0.0375 Mpa and 0.085 Mpa respectively. In the same manner, Yeoh C1 coefficients were 0.27 Mpa, 0.07 Mpa and 0.056 Mpa (S1 Appendix). Furthermore, elastic properties were provided to the pelvic floor muscles and fasciae. The Poisson coefficients set were 0.45 for all, and a Young Modulus of 0.1 Mpa for the pelvic floor and 0.18 Mpa for the fasciae (S1 Appendix). Regarding the corrective filament structures, we used elastic properties with high stiffness properties and a constant beam section to mimic the behavior of the tread.

We focused on the interactions between each part while taking many assumptions into consideration for an accurate organ mobility simulation. At this step, the presence of fascia as connective tissues between the main organs (vagina, rectum, bladder) shows there is no direct contact between them which means the necessity of setting interaction properties accordingly [31]. Moreover, we defined the interactions between the main organs (vagina, rectum, bladder) as the master interaction, and the fasciae and pelvic floor surrounding them as the slave setting. By choosing this configuration and redefining the mesh of the slave parts as tetrahedral, we ensured that the calculations of these interactions remained possible and performed by the software while keeping the highest precision.

Regarding the boundary conditions, we fixed the pelvic floor borders closed to the bone and ligament structures as an Encastre element. The area of the ischial spine was free while the sacrum and coccyx were also fixed as Encastre elements. This setting permitted to simulate the proper support given by the bone structures to the pelvic floor and hence the rest of the key organs under study.

To simulate the patient specific organ displacement seen on the MRI analysis and estimate the force exerted by the abdominal pressure on the pelvic organs, two different forces were applied. One on the upper surface of the vagina with a downward vector direction and one on the top surface of the bladder with 45 degrees from the sagittal axis in an anteroposterior direction. We considered this direction of pressure to be the most similar to the one naturally exerted by the patient as seen in the dynamic MRI images but also taking into account the preliminary results shown in Mayeur et al. study [25]. The pressure values were applied as surface traction load and increased empirically until we measured the same displacement on the same key node elements (C, Ba and Bp) relative to the MRI. We used the query node tool to measure the exact node displacement.

At this stage of the FE modulization, the number of elements and mesh definition was chosen after mesh convergence to ensure the stability of the calculations and accuracy of the results. We obtained a model with 11.000 elements, divided into 3.500 shell (S3-S4) and 7.500 volume (C3D10). S3 elements are commonly used for structural thin shell elements with 3 triangular nodes and S4R for structural thin elements with 4 triangular nodes for hourglass control and constant pressure. Shell was taken into consideration for each organ and muscle localization (S2 Appendix). C3D10H is commonly used for structural solid sections with hybrid 10 tetrahedral node elements, used here for fasciae and cavities volumes.

In order to measure pelvic organ mobility on dynamic MRI and compared it to the Finite Element results, we used the pubococcygeal line (PCL) methodology [30]. The PCL is drawn from the inferior border of the pubic symphysis to the last coccygeal joint (Fig 2). The perpendicular distance from the reference points to the PCL was measured both at rest and at maximal strain, usually during the defecation phase. In the anterior compartment, the reference point (Ba point) was the most posterior and inferior aspect of the bladder base. In the middle compartment, the reference point (C point) was the cervix’s most anterior and inferior aspect or the vaginal apex (in case of total hysterectomy). In the posterior compartment, the anterior aspect of the anorectal junction was the reference point (Bp point). All measures were expressed in mm.

To further study the pelvic organ mobility with the FE model, we used the same pubococcygeal method. With the FE model, we firstly compared a right and/or left posterior SSF. In this first phase, the distance between the anchorage zone and the ischial spine was 2cm. Then, different distances between the anchorage zone on the sacrospinous ligament and the ischial spine were studied: 1 cm, 2 cm and 3 cm.

Outcomes measures were the displacement during maximal strain versus rest of the anterior reference point (Ba), the apical reference point (C) and the posterior reference point (Bp) according to the PCL method on the midsagittal plane.

## Results

### Generic model

**Fig 4:**
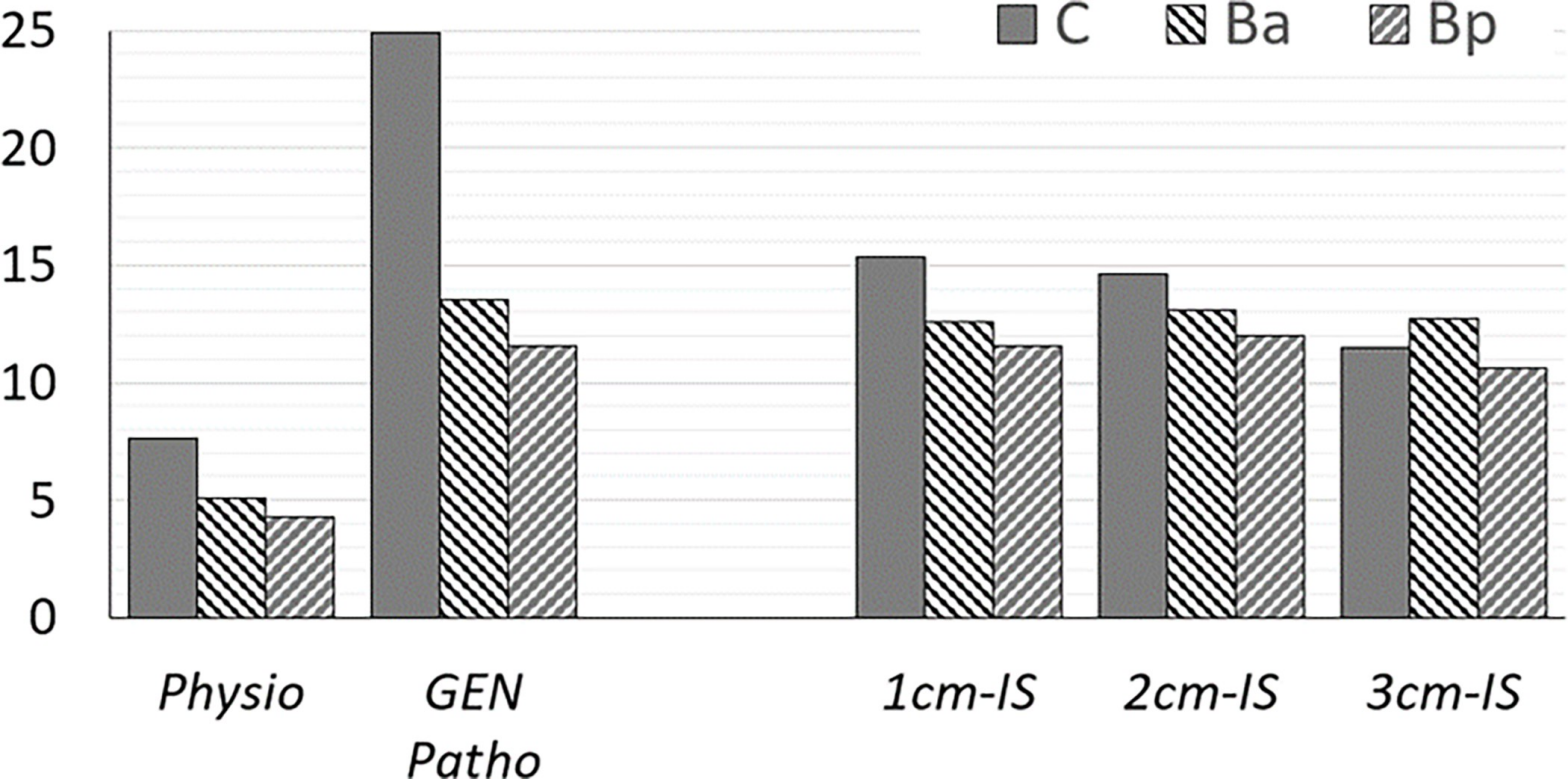
Displacement study of C, Ba and Bp points (mm) under physiological and pathological conditions or according to the distance between the anchorage zone on the sacrospinous ligament and the ischial spine (1 or 2 or 3 cm). IS: Ischial spine; physio: Physiological conditions; GEN patho: Pathological conditions.

According to the generic model, C point was displaced by 7 mm under physiological conditions (without simulation of a pelvic organ prolapse) versus 25 mm under pathological conditions (Fig 4). The anterior vaginal wall (Ba point) was displaced by 5 mm and 13 mm under physiological and pathological conditions, respectively. The posterior vaginal wall (Bp point) was displaced by 4 mm under physiological and 12 mm under pathological conditions.

Overall, pelvic organ mobility decreased after surgery, regardless of the surgical technique. C point was displaced by 14.1 mm, 14.1 mm and 11.5 mm after left, right and bilateral SSF respectively. Ba point was displaced by 12.7 mm, 12.7 mm and 12 mm after left, right and bilateral SSF respectively. Bp point was displaced by 10.6 mm, 10.6 mm and 9.9 mm after left, right and bilateral SSF respectively.

C point was displaced by 15.4 mm, 14.6 mm and 11.5 mm when the suture on the sacrospinous ligament was performed at 1 cm, 2 cm and 3 cm from the ischial spine respectively in the configuration of bilateral posterior sacrospinous ligament fixation. Ba point was displaced by 12.6 mm, 13.1 mm and 12.9 mm when the suture on the sacrospinous ligament was performed at 1 cm, 2 cm and 3 cm from the ischial spine respectively. Bp point was displaced by 11.6 mm, 12.0 mm and 10.6 mm when the suture on the sacrospinous ligament was performed at 1 cm, 2 cm and 3 cm from the ischial spine respectively.

### Patient-specific model

Preoperatively, pelvic organ displacement was similar between the dynamic MRI and the patient-specific Finite Element model. The C, Ba and Bp points were displaced by 7 mm, 29 mm and 6 mm, respectively, between the resting sequence and the maximum strain sequence.

**Fig 5:**
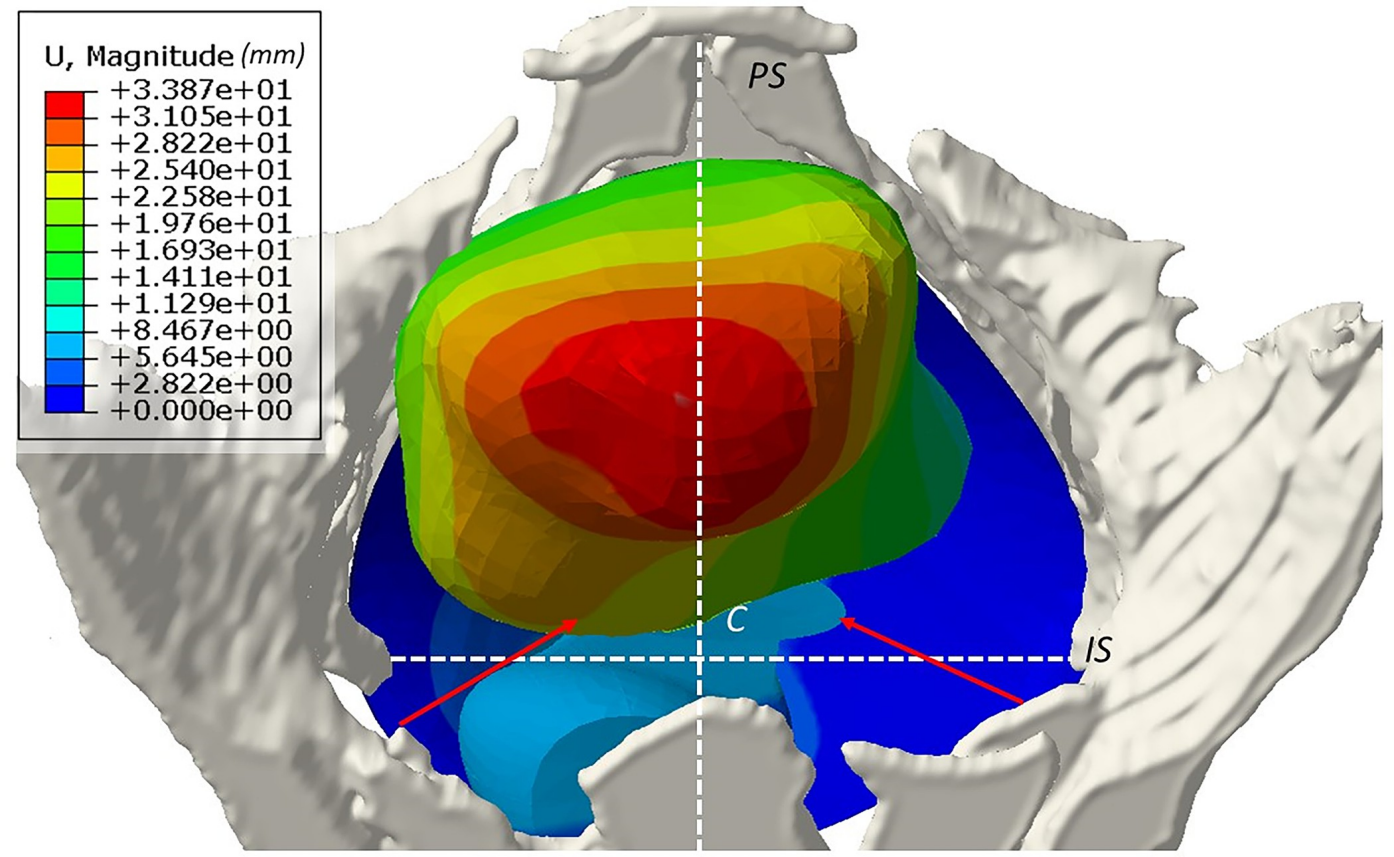
Asymmetrical pelvic organ mobility according to the patient-specific finite element model before surgery (top anatomical view). Red arrows: Scheme of the sutures between the vaginal apex and the ischial spine. Colormap: Small displacements in blue and maximum displacements in red.

During the construction of the patient-specific model and before the surgery simulation, a significative and asymmetric organ displacement of the bladder was observed on the x-axis (Fig 5). Therefore, the displacement of Ba point during surgery simulation could not be analyzed.

**Fig 6:**
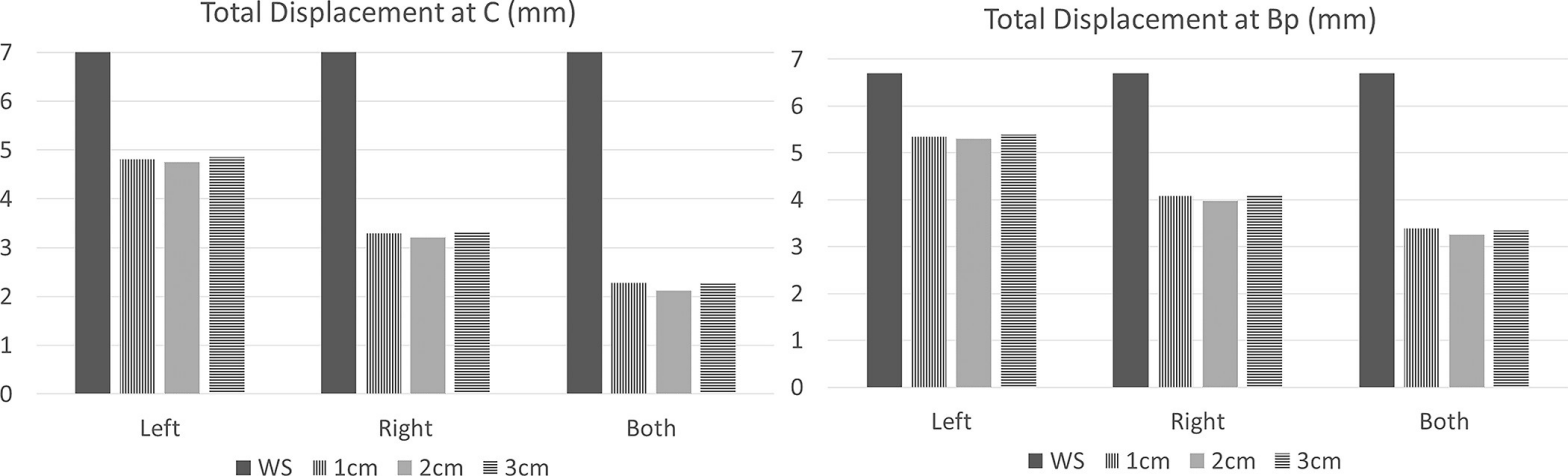
Study displacement (mm) of C and Bp points according to a left and/or right SSF and the distance between the anchorage zone and the ischial spine (1cm, 2 cm or 3cm). WS: Without surgery, both: Bilateral SSF.

Without surgery, total displacement of C point and Bp point was about 7 mm and 6.5 mm respectively. C point was displaced by 4.74 mm, 3.21 mm and 2.12 mm and Bp point by 5.30 mm, 3.98 mm and 3.24 mm after left, right and bilateral posterior SSF respectively (Fig 6).

C point was displaced by 4.80 mm, 4.75 mm and 4.85 mm and Bp point by 5.35 mm, 5.30 mm and 5.38 mm when the suture on the left sacrospinous ligament was performed at 1 cm, 2 cm and 3 cm from the ischial spine, respectively. Similarly, Bp et C points were similarly displaced when the suture on the right or both sacrospinous ligament was performed at 1 cm, 2 cm and 3 cm from the ischial spine.

## Discussion

According to the generic model, pelvic organ mobility appeared similar after right or left posterior SSF. The vaginal apex mobility appeared to decrease when the suture was bilateral or away from the sciatic spine. The mobility of the anterior vaginal wall (Ba point) was similar regardless of the distance between the suture and the ischial spine.

According to the patient-specific model, the vaginal apex and the rectum mobilities were reduced after posterior SSF, regardless the surgical procedure. Organ displacements appeared greater during left SSF than during right and bilateral SSF. Moreover, organ mobility was reduced in all measurement points during bilateral SSF. The distance between the anchorage zone and the ischial spine did not appear to have a significant impact on organ displacement.

Our study suggested that the best approach was to suture the vaginal apex bilaterally. If a single suture is preferred, it appears that organ mobility is reduced on the right sacrospinous ligament. A bilateral fixation should provide an additional support by improving the ability of the vagina to withstand increased intra-abdominal pressure [32]. In the literature, there are only two studies comparing unilateral and bilateral posterior sacrospinous ligament fixation. Salman et al. showed that unilateral and bilateral sacrospinous ligament fixation produced similar clinical outcomes [13]. But, the follow-up was short (6 months) and the number of patients was small. In the study by Jones et al., anatomical success rates for both procedures were similar (90.2% women in the unilateral SSF group versus 85.5% in the bilateral SSF group; p  =  0.56) [33].

According to the generic model, the mobility of the apical point seemed to be reduced when the suture was 3 cm from the ischial spine. We were unable to confirm this fact with the patient-specific model. However, the anatomy of the sacrospinous ligament must be considered. It is a complex anatomical region. Fixation must avoid neurovascular structures. According to Giraudet et al., the vaginal apex should be sutured 2 cm from the ischial spine to reduce nerve and vascular injuries [14].

In the literature, there are no studies evaluating biomechanically the SSF. But, Hachenderg et al. evaluated the biomechanical difference between an in-line sacrospinous ligament suture versus an orthogonal ligament suture [34]. In their in-vitro model, an orthogonal suture displayed a significantly higher ultimate load than an in-line suture (80N versus 57 N, p<0.05).

It is well known that a posterior sacrospinous ligament fixation tends to deviate the vaginal apex downwards leaving the anterior compartment more vulnerable to recurrent POP [35]. A bilateral (anterior and posterior) approach could resolve the dorsal-caudal deviation of the vaginal axis and offer a concomitant anterior repair and sore symmetrical vaginal cavity [32,36]. It could be the next step of our work.

Our study is the first of its kind. Generic model of POP physiopathology or POP repair already exist in the literature [16,17,26]. But, no study has currently evaluated the mobility and stresses of the pelvic organs before and after apical prolapse repair by posterior sacrospinous ligament fixation. Moreover, these studies were not patient-specific [25,26]. Patient-specific model are personalized model according to the patient’s pre-operative MRI. The originality of this work is that we also studied this surgery with a patient-specific model. This model was parameterized according to the patient’s radiological characteristics in order to personalize the management of POP. These primary results need to be confirmed by more powerful clinical and mechanical studies involving a greater number of patients.

The mobility of the anterior wall (Ba point) was not studied with the patient-specific model because its large displacement compared to the displacement of the other measurements points and its asymmetrical displacement was extremely difficult to accurately simulate (29 mm compared to 7 mm of point C). If the large and accurate displacement at Ba point had been simulated, our results on the other measurement points would have been affected to the point of not obtaining patient-specific results. Therefore, we preferred to keep as many points with a validated patient-specific organ displacement such as C and Bp points as possible rather than having only point validated (Ba point).

Another limit of the patient-specific model was the non-concordance between the clinical examination and the dynamic MRI results. Indeed, the stage III apical prolapse of the patient was not observed during the dynamic pelvic MRI. Obringer et al. also found that the correlation between clinical scores and MRI was 53.3% only [37]. This fact must be considered when assessing simulated surgical techniques based on dynamic MRI.

We managed to reproduce the patient specific conditions and organ motion into the FE model simulation while respecting the direction and total displacement of pelvic organs as measured on the dynamic pelvic MRI. This important step allows us to discover new methods and find solutions for prolapse surgery and organs displacement cure. From the patient-specific model, it is possible to perform a series of surgical techniques and compare them to decide which option is the most effective for the patient’s needs and anatomical specifications.

## Conclusion

According to the generic model and the patient-specific model, the vaginal apex appeared to be less mobile in bilateral posterior SFF. The anchorage area on the sacrospinous ligament appeared to have little effect on the pelvic organ mobilities. The generic model could be useful for predicting the anatomical outcomes of posterior SSF. In addition, the patient-specific model seemed to identify excessive mobility of the pelvic floor organs. It appears to be an interesting tool for assessing the best anchorage area selection. The patient-specific model is likely to be the future of surgery in order to personalize POP surgery and improve surgical success rates. Nevertheless, further research is required to ensure that the results are clinically acceptable.

## Supporting information

S1 Appendix Table values for the anatomical structures containing element type, thickness, element number, and material properties for linear elasticity (Young’s Modulus) and hyperelastic models (Yeoh’s coefficients). (PDF)

S2 Appendix Patient-specific finite element (FE) model with loading conditions, boundary conditions (BC) and mesh definition. Sagittal (a) and posterosuperior (b) views of the patient-specific FE model. The red surfaces are surfaces on which the pressure was applied. The force orientation is shown by the arrows. The blue and orange triangles are the boundaries conditions fixing the pelvic floor. Posterosuperior (c) and sagittal (d) views of the volume mesh.
(TIF)
