# Supplementary material for: Mobility analysis of a posterior sacrospinous fixation using a finite element model of the pelvic system
Source: PLoS One. 2024 Mar 21;19(3):e0299012. doi: 10.1371/journal.pone.0299012 (PMC10956756; doi:10.1371/journal.pone.0299012)
Supplement: S1 Appendix — (PDF) [file pone.0299012.s001.pdf]

| **Anatomical Structure** | **Element Type** | **Thickness (mm)** | **Element Number** | **Material properties *(Young's Modulus)*** | **Material properties *(Yeoh's model)*** | |
|---|---|---|---|---|---|---|
| | | | | E (MPa) | C0 (MPa) | C1 (MPa) |
| Vagina (Vag) | S4R/S3 | 3 | 400 | na | 0,111 | 0,27 |
| Bladder (Bla) | S4R | 2 | 800 | na | 0,0375 | 0,07 |
| Rectum (Rec) | S4R | 2 | 400 | na | 0,085 | 0,0565 |
| Cavity volumes | C3D10h | na | 6000 | na | na | na |
| Pelvic Floor (PF) | S4R/S3 | 2 | 2000 | 0,1 | na | na |
| Bla/Vag Fascia | C3D10 | na | 800 | 0,18 | na | na |
| Vag/Rec Fascia | C3D10 | na | 300 | 0,18 | na | na |
| Rec/PF Fascia | C3D10 | na | 300 | 0,18 | na | na |
