# Supplementary figures and images for: Mobility analysis of a posterior sacrospinous fixation using a finite element model of the pelvic system
Source: PLoS One. 2024 Mar 21;19(3):e0299012. doi: 10.1371/journal.pone.0299012 (PMC10956756; doi:10.1371/journal.pone.0299012)

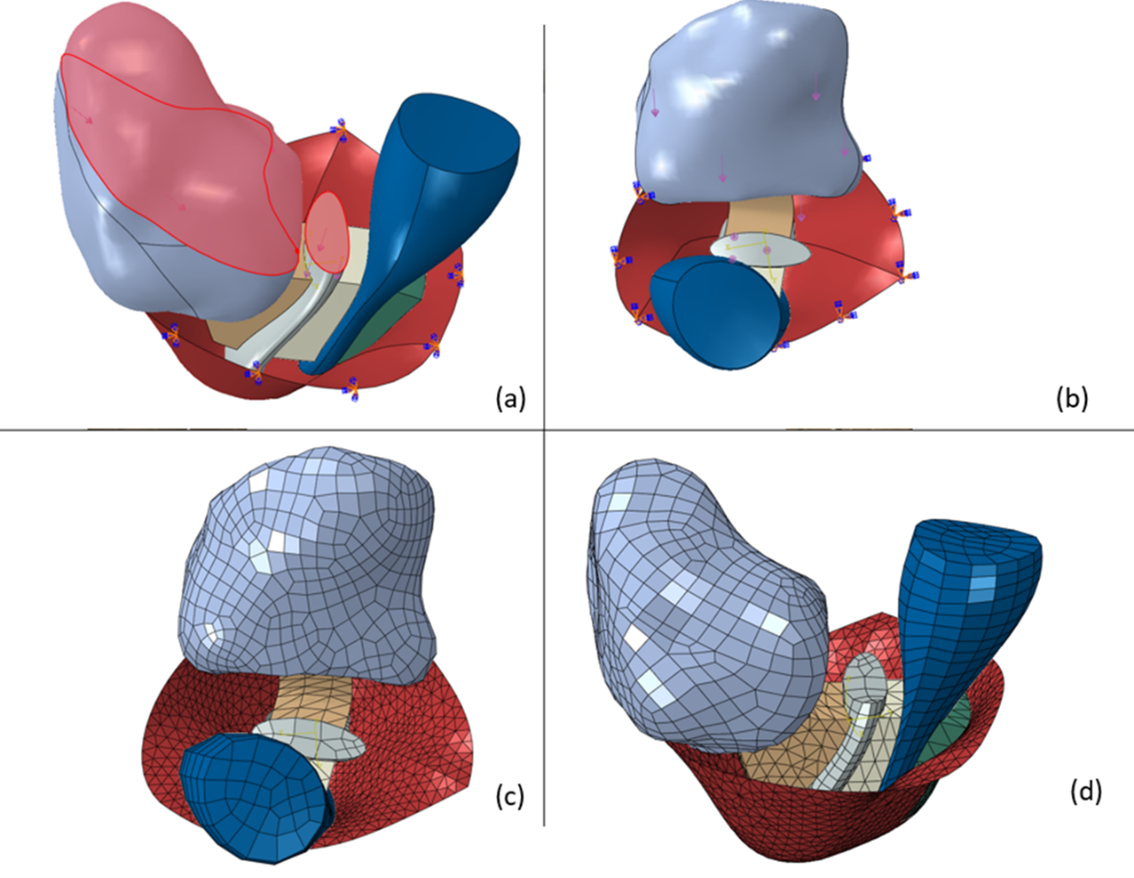

Supplement: S2 Appendix — Sagittal (a) and posterosuperior (b) views of the patient-specific FE model. The red surfaces are surfaces on which the pressure was applied. The force orientation is shown by the arrows. The blue and orange triangles are the boundaries conditions fixing the pelvic floor. Posterosuperior (c) and sagittal (d) views of the volume mesh. (TIF) [file pone.0299012.s002.tif]
